# Parkinson’s Disease With Depression: The Correlations Between Neuroinflammatory Factors and Neurotransmitters in Cerebrospinal Fluid

**DOI:** 10.3389/fnagi.2020.574776

**Published:** 2020-10-23

**Authors:** Teng-hong Lian, Peng Guo, Ya-nan Zhang, Jing-hui Li, Li-xia Li, Du-yu Ding, Da-ning Li, Wei-jiao Zhang, Hui-ying Guan, Xiao-min Wang, Wei Zhang

**Affiliations:** ^1^Department of Neurology, Beijing Tiantan Hospital, Capital Medical University, Beijing, China; ^2^Department of Blood Transfusion, Beijing Tiantan Hospital, Capital Medical University, Beijing, China; ^3^Department of General Internal Medicine, Beijing Tiantan Hospital, Capital Medical University, Beijing, China; ^4^Department of Physiology, Capital Medical University, Beijing, China; ^5^Center for Cognitive Neurology, Department of Neurology, Beijing Tiantan Hospital, Capital Medical University, Beijing, China; ^6^China National Clinical Research Center for Neurological Disease, Beijing Tiantan Hospital, Capital Medical University, Beijing, China; ^7^Center of Parkinson’s Disease, Beijing Institute for Brain Disorders, Beijing, China; ^8^Beijing Key Laboratory on Parkinson Disease, Beijing, China

**Keywords:** Parkinson’s disease, depression, neuroinflammatory factors, neurotransmitters, cerebrospinal fluid

## Abstract

**Background**: To explore the changes of neuroinflammatory factors in cerebrospinal fluid (CSF) and their correlation with monoamine neurotransmitters in Parkinson’s disease (PD) with depression (PD-D) patients.

**Methods**: Neuroinflammatory factors and neurotransmitters in CSF were measured and compared between PD with no depression (PD-ND) and PD-D groups. The relationship between PD-D and neuroinflammatory factors was studied by binary logistic regression equation, and the related factors of PD-D were adjusted. The correlations of the levels of neuroinflammatory factors and neurotransmitters in PD-D group were analyzed.

**Results**: The levels of tumor necrosis factor (TNF)-α in CSF from PD-D group were significantly higher and there were no significant differences in the levels of interleukin-1β, prostaglandin (PG) E_2_, hydrogen peroxide (H_2_O_2_), and nitric oxide (NO). The 24-item Hamilton Depression Scale (HAMD-24) score was positively correlated with the level of TNF-α in CSF. Binary logistic regression showed that the OR of CSF TNF-α level was 1.035 (95% CI 1.002–1.069). The level of dopamine (DA) in CSF of PD-D group was significantly lower than that in PD-ND group. TNF-α level was negatively correlated with DA level in CSF from PD patients (*r* = −0.320, *P* = 0.003).

**Conclusions**: Neuroinflammatory factors, especially TNF-α, may play an important role in PD-D. It may cause damage to DA neurons and lead to the depletion of DA, which is related to the occurrence and development of PD-D.

## Introduction

Parkinson’s disease (PD) is the second most common neurodegenerative disorder with a number of characteristic motor symptoms, including rest tremor, rigidity, bradykinesia, and gait and postural instabilities (Jankovic, 2008). Additionally, PD patients also have a variety of non-motor symptoms, such as mood abnormality, sleep disturbance, autonomic dysfunction, cognitive impairment, etc. Depression is one of the most common non-motor symptoms of PD (Cosentino et al., 2013). Compared with healthy controls, PD with depression (PD-D) patients displayed a higher morbidity, which was 40% at the early stage and reached up to 70% at advanced stage of disease (Reijnders et al., 2008; Cosentino et al., 2013). However, both diagnosis and treatment of PD-D have not been given enough attention for patients. The failures to diagnose PD-D and offer timely treatment significantly compromise the life quality of both PD patients and their caregivers (Goodarzi et al., 2016). Importantly, the underlying mechanisms of PD-D are not clearly elucidated, and thus effective therapy for PD-D patients still needs to be developed.

Increasing studies showed that primary depression was closely related to inflammation (Kohler et al., 2016). In the clusters of inflammatory markers measured, C-reactive protein (CRP) level in plasma was found to be significantly correlated with the severity of depressive symptoms (Felger et al., 2020). Furthermore, repetitive transcranial magnetic stimulation exerted an anti-depressant effect *via* an anti-inflammation mechanism in a mouse model of primary depression (Tian et al., 2020), indicating the pivotal role of inflammation on primary depression.

It can be suspected that inflammation probably also plays a role on the depression caused by neurodegenerative disease, like PD. We know that it is more meaningful to investigate brain neuroinflammation by detecting the inflammatory factors in cerebrospinal fluid (CSF) than peripheral inflammation by measuring the inflammatory factors in plasma from PD-D patients. Although non-motor symptoms of PD, including cognitive impairment (Sanjari Moghaddam et al., 2018), pure apathy (Wang et al., 2016) and sleep disorders (Wang et al., 2016), particularly rapid eye movement sleep behavior disorder (RBD) (Hu et al., 2015), were significantly correlated with multiple neuroinflammatory factors in CSF demonstrated by our and other investigations, there were very few studies about the relationship between PD-D and brain neuroinflammation. For example, Lindqvist et al. (2013) found that the more severe depression was significantly associated with the higher CRP level in CSF from 71 non-demented PD patients. However, there are no studies in large samples with more neuroinflammatory factors in CSF from PD-D patients measured and analyzed.

It is known that the alterations of neurotransmitters may serve as the biochemical basis of PD-D. The dysfunctions of dopamine (DA; Lian et al., 2018), 5-hydroxytryptamine (5-HT; Zhuo et al., 2017; Lian et al., 2018), and norepinephrine (NE; Zhuo et al., 2017) systems as well as their interaction in the brain were related to PD-D. Through analysis of neurotransmitters in CSF from PD patients, our previous study showed that, compared with 5-HT and NE, only DA level in PD-D group was significantly reduced compared with that in PD with no depression (PD-ND) group. Moreover, the score of the 24-item Hamilton Depression Scale (HAMD-24) had a negative correlation only with DA level in CSF (Lian et al., 2018). Therefore, DA may play a more important role in PD-D. Additionally, it was found that alleviating inflammation prevented dopaminergic neurodegeneration (Chen et al., 2019) and increased DA level (Wang et al., 2015) in a mouse PD model. However, there was no investigation exploring the correlations of neuroinflammatory factors with neurotransmitters in CSF from PD-D patients.

In this study, HAMD-24 was used to assess the depressive symptoms of PD patients; the levels of neuroinflammatory factors, including hydrogen peroxide (H_2_O_2_), nitric oxide (NO), tumor necrosis factor (TNF)-α, IL (interleukin)-1β, IL-6, prostaglandin (PG)E_2_, and neurotransmitters, including DA, 5-HT, and NE in CSF, were measured and compared between PD-D and PD-ND groups; the correlations among the score of HAMD-24, the levels of neuroinflammatory factors, and neurotransmitters in PD-D group were analyzed.

## Materials and Methods

### Participants

#### PD Patients

PD patients were consecutively recruited according to the 2015 International Parkinson and Movement Disorders Society (MDS) diagnostic criteria for PD (Postuma et al., 2015).

This study was approved by Beijing Tiantan Hospital ethics committee. Written informed consents were obtained from all participants in this study.

#### PD-D and PD-ND Patients

Inclusion criteria: PD-D was established by the diagnostic criteria for PD with depression (Starkstein et al., 2011). HAMD-24 recommended by PD-D diagnostic criterion for assessing depressive symptoms of PD includes seven factors: anxiety/somatization, weight loss, cognitive impairment, circadian fluctuations, retardation, sleep disturbances, and hopelessness, by which the clinical characteristics of depressive patients are clearly and explicitly reflected. PD patients with a total score of HAMD-24 ≥8 points were assigned to PD-D group, in which 8–19 points, 20–34 points, and ≥35 points were categorized as mild, moderate, and severe depression, respectively. PD patients with a total score of HAMD-24 < 8 points were assigned to PD-ND group.

Exclusion criteria: PD patients who met the following criteria were excluded: (1) other central nervous systemic diseases; (2) severe systemic diseases; (3) depression caused by organic mental disorders, psychoactive substances, addictive substances, etc.; (4) infections and immune diseases; and (5) usage of anti-depressants.

### Clinical Assessments for PD Patients

A total of 86 PD patients were consecutively recruited from the Beijing Tiantan Hospital, Capital Medical University, at the baseline, among which 58 cases met the PD-D inclusion and exclusion criteria and the remaining 28 cases were with PD-ND.

### Collection of Demographic Information

Medical information, including gender, age, age of onset, duration of disease, side of onset, education level, and anti-PD therapy, including levodopa equivalent daily dose (LEDD), the types of drugs, the duration of taking drugs, etc., were collected.

### Assessment of Motor Function

Disease severity: the severity of PD was assessed by using the Hoehn-Yahr (H-Y) stage.

Motor symptoms: the motor symptoms of PD patients, including tremor, bradykinesia, rigidity, and postural and gait abnormalities, were assessed during the “off” period. The higher the total score of UPDRS III, the worse the motor symptoms.

Motor phenotypes: according to Jankovic’s clinical phenotype classification, PD patients were divided into three motor phenotypes, including tremor-dominant (TD), postural instability and gait difficulty (PIGD), and mixed types (Jankovic et al., 1990). Tremor was assessed by item 16, 20, and 21, and PIGD was assessed by item 13, 14, 15, 29, and 30 in UPDRS III. Motor phenotype was determined by the ratio of average tremor score and PIGD score, and the ratio of ≥1.5, ≤1.0, and 1.0–1.5 were identified as TD, PIGD, and mixed types, respectively.

### Assessments of Non-motor Symptoms

Non-motor symptoms: cognitive impairment was assessed by the Montreal Cognitive Assessment Scale (MoCA; Hoops et al., 2009); anxiety was assessed by the 14-item Hamilton Anxiety Scale (HAMA-14; Hamilton, 1959); fatigue was assessed by the 14-item Chalder Fatigue Scale (FS-14; Chalder et al., 1993); RBD was assessed by the RBD Screening Questionnaire (RBDSQ; Zea-Sevilla and Martínez-Martin, 2014); autonomic dysfunction was assessed by the Scale for Outcomes in PD for Autonomic Symptoms (SCOPA-AUT; Visser et al., 2004); restless legs syndrome (RLS) was assessed by RLS Severity Rating Scale (RLSRS; Walters et al., 2003).

### Assessment of the Activities of Daily Living (ADL)

ADL, including basic ADL (BADL) and instrumental ADL (IADL), was assessed by the ADL Scale (Holroyd et al., 2005).

### Measurements of Neuroinflammatory Factors and Neurotransmitters in CSF

#### Collection and Processing of CSF

Anti-PD drugs were withdrawn for 12–14 h if the patients’ condition allowed and longer time was considered unethical by our ethical committee. Because the medical washout period should be three times the *t*_1/2_ of the anti-PD drugs, the subjects taking drugs with long *t*_1/2_, including controlled release Sinemet (Sinement CR), Pramipexole, and controlled-release Piribedil (Piribedil CR), were excluded in the analyses of neurotransmitters.

Under fasting conditions, 5 ml of CSF was taken in a polypropylene tube through lumbar puncture between 7 and 9 a.m. CSF samples were centrifuged immediately at 3,000 rpm at 4°C. Then, approximately 0.5-ml volume of CSF was aliquoted into separate Nunc cryotubes and kept frozen at −80°C until usage in the assays (Lian et al., 2018).

#### Measurements of Neuroinflammatory Factors in CSF

The levels of H_2_O_2_ and NO in CSF from PD patients were measured by the A064 assay kit and A012 assay kit (Jiancheng Bioengineering Institute, Nanjing, China), respectively, by the chemical colorimetric method (Wang et al., 2016).

The levels of TNF-α, IL-1β, IL-6 and PGE_2_ in CSF from PD patients were measured by the enzyme linked immunosorbent assay. Kit 1R040, 1R350, and D6050 (R&D Systems parent company, USA) were used for TNF-α, IL-1β, and IL-6, respectively. Kit CSB-E07965h (CUSABIO Technology Limited Liability Company, USA) was used for PGE_2_.

#### Measurements of Neurotransmitters in CSF

The levels of DA, 5-HT, and NE in CSF from PD patients were measured by high-performance liquid chromatography (Lian et al., 2018). LC-MS-MS 6410 chromatograph and Phenomenex 150 × 2 mm and 150 × 3 mm chromatographic columns were from the Agilent Company (California, USA), and the standard sample was from Sigma Company (California, USA).

### Statistical Analyses

Statistical analyses were performed by using SPSS Statistics 20.0. A *P*-value of less than an alpha level of 0.05 was defined as statistically significant.

The levels of neuroinflammatory factors in CFS between PD-D and PD-ND groups were compared. Normal distributed measurement data were presented as means ± standard deviations, and non-normal distributed measurement data were presented as median (first quartile, third quartile). In the analyses between PD-D and PD-ND groups, those that meet the normal distribution and the homogeneity of variance were compared by *t* test; the rest of the data were compared by Mann–Whitney *U* test.

Bivariate correlation analysis was performed between HAMD-24 score and the level of neuroinflammatory factors in CSF from PD patients.

Binary logistic regression analysis was used to investigate the relationship between PD-D and the level of neuroinflammatory factors in CSF. In the binary logistic regression equation, neuroinflammatory factors in CSF with statistical differences in single-factor analysis and in the bivariate correlation analysis were respectively set as covariates and put into multi-logistic regression models, whereas with or without depression in PD was set as a dependent variable (PD-D = 1, PD-ND = 0).

## Results

### Demographic Variables, Motor Function, Non-motor Symptoms, and ADL of PD-D and PD-ND Groups

Demographic variables, the scores of motor symptoms, non-motor symptoms, and ADL were compared between PD-D and PD-ND groups (Table 1). The data displayed that PD-D group had significantly lower education level, higher UPDRS III score, greater proportion of PIGD type, higher scores of HAMA, FS, RBDSQ, and SCOPA-AUT scales, and lower score of ADL scale than PD-ND group (*P* < 0.05).

**Table 1:** Demographic variables, scores of motor function, non-motor symptoms, and ADL in PD with depression (PD-D) and PD with no depression (PD-ND) groups; **P < 0.01, *P < 0.05.

	PD-ND group (28 cases)	PD-D group (58 cases)	*P*
Demographic variables			
Female (*n*, %)	13 (46.43%)	27 (46.55%)	0.991
Age [year, median (Q1–Q3)]	60.50 (51.75–70.00)	60.00 (56.00–68.00)	0.938
Age of onset (year, $\bar{\text{X}}$ ± s)	57.39 ± 10.76	56.44 ± 9.30	0.676
Disease duration [year, median (Q1–Q3)]	3.00 (2.00–5.00)	3.00 (1.50–5.00)	0.683
Low education level (<9 years; *n*, %)	7 (25.00%)	28 (48.28%)	0.040*
Side of onset (*n*, %)	14 (50.00%)	26 (44.83%)	0.652
LEDD
Motor function			
H–Y stage (*n*, %)			
Early stage (stage 1–2.5)	23 (82.14%)	51 (87.93%)	0.515
Advanced stage (stage 3–5)	5 (17.86%)	7 (12.07%)	
Total UPDRS III score [point, median (Q1–Q3)]	16.00 (12.00–29.25)	29.50 (20.75–42.00)	0.001**
Tremor	3.00 (2.00–7.50)	5.00 (3.00–7.00)	
Rigidity	2.00 (0.25–4.75)	5.00 (2.00–8.25)	
Bradykinesia	6.50 (4.25–12.00)	12.00 (8.00–17.00)	
Postural instability/gait difficulty	2.50 (2.00–4.00)	4.00 (2.00–6.00)	
Motor phenotypes (*n*, %)			
PIGD type	11 (39.29)	55 (94.83%)	<0.001**
TD type	6 (21.43%)	3 (5.17%)	
Mixed type	11 (39.29%)	0 (0.00%)	
Non-motor symptoms			
MoCA [point, median (Q1–Q3)]	21.50 (17.00–24.00)	18.50 (14.50–25.00)	0.631
HAMA-14 [point, median (Q1–Q3)]	2.00 (1.00–3.75)	10.00 (7.00–17.5)	<0.001**
FS-14 [point, median (Q1–Q3)]	7.00 (3.25–9.50)	10 (7.00–12.00)	0.003**
RBDSQ [point, median (Q1–Q3)]	1.00 (0.00–4.00)	4.00 (1.00–5.50)	0.018*
SCOPA-AUT [point, median (Q1–Q3)]	31.50 (28.25–36.25)	35.50 (31.00–40.75)	0.004**
RLSRS [point, median (Q1–Q3)]	0.00 (0.00–8.00)	0.00 (0.00–15.25)	0.108
ADL			
ADL [point, median (Q1–Q3)]	23.00 (20.00–30.75)	32.00 (23.75–43.00)	0.003**

### Comparisons of Neuroinflammatory Factors in CSF Between PD-D and PD-ND Groups

The levels of neuroinflammatory factors, including H_2_O_2_, NO, TNF-α, IL-1β, IL-6, and PGE_2_ in CSF from PD-D and PD-ND groups, were compared (Table 2). The results showed that TNF-α level in CSF from PD-D group was significantly increased compared with that from PD-ND group (31.321 pg/ml vs. 21.530 pg/ml, *P* < 0.05). However, there was no significant difference in the levels of H_2_O_2_, NO, IL-1β, IL-6, and PGE_2_ in CSF between the two groups.

**Table 2:** Levels of neuroinflammatory factors in cerebrospinal fluid (CSF) from PD-D and PD-ND groups; *P < 0.05.

	PD-ND group (28 cases)	PD-D group (58 cases)	*P*
H_2_O_2_ [mmol/L, median (Q1–Q3)]	3.342 (1.931–12.532)	2.580 (1.973–9.772)	0.311
NO [mmol/L, median (Q1–Q3)]	53.892 (42.784–75.000)	52.821 (35.682–74.603)	0.839
TNF-α [pg/ml, median (Q1–Q3)]	21.530 (17.054–33.689)	31.321 (19.682–73.178)	0.027*
IL-1β [pg/ml, median (Q1–Q3)]	12.401 (10.297–22.112)	17.078 (11.079–23.522)	0.394
IL-6 [pg/ml, median (Q1–Q3)]	3.436 (1.885–5.552)	3.498 (1.885–5.802)	0.749
PGE_2_ [pg/ml, median (Q1–Q3)]	13.096 (3.692–16.004)	10.043 (5.540–15.621)	0.847

*H_2_O_2_, hydrogen peroxide; NO, nitric oxide; TNF-α, tumor necrosis factor-α; IL-1β, interleukin-1β; IL-6, interleukin-6; PGE_2_, prostaglandin E_2_*.

### The Relationship Between PD-D and Neuroinflammatory Factors in CSF

The correlation between the score of HAMD-24 and the levels of H_2_O_2_, NO, TNF-α, IL-1β, IL-6, and PGE_2_ in CSF from PD patients was analyzed (Table 3). It was found that HAMD-24 score had a positive correlation with TNF-α level in CSF (*r* = 0.247, *P* = 0.022). However, no significant relationship between HAMD-24 score and the levels of H_2_O_2_, NO, IL-1β, IL-6, and PGE_2_ in CSF was observed between the two groups.

**Table 3:** Correlation between the score of 24-item Hamilton Depression Scale (HAMD-24) and the levels of neuroinflammatory factors in CSF from PD patients; *P < 0.05.

	*R*	*P*
H_2_O_2_	−0.176	1.704
NO	−0.016	0.882
TNF-α	0.247	0.022*
IL-1β	0.004	0.970
IL-6	0.145	0.260
PGE_2_	0.068	0.534

*H_2_O_2_, hydrogen peroxide; NO, nitric oxide; TNF-α, tumor necrosis factor-α; IL-1β, interleukin-1β; IL-6, interleukin-6; PGE_2_, prostaglandin E_2_*.

Binary logistic regression analysis was further performed to investigate the correlation between PD-D and TNF-α level in CSF (Table 4). The results suggested that the OR of TNF-α level was 1.035, and the 95% CI was 1.003–1.069 (*P* < 0.05) after the adjusting the risk factors of PD-D, including PIGD type, and the scores of HAMA-24 and UPDRS III scales observed in our previous study.

**Table 4:** Binary logistic regression analysis of TNF-α in CSF from PD-D group; *P < 0.05.

	*B*	OR	95% CI	*P*
HAMA-24 score	0.032	1.033	0.968–1.102	0.333
PIGD type	0.478	1.613	1.176–2.213	0.003
UPDRS III score	−2.573	0.076	0.008–0.75	0.027
FS-14 score	0.007	1.007	0.748–1.356	0.962
TNF-α level in CSF	0.034	1.035	1.002–1.069	0.039*
Constant number	−3.429	0.032		0.023

*The model corrects HAMA score, PIGD type, UPDRS III score, and FS score. HAMA-14, 14-item Hamilton Anxiety Scale; PIGD, postural instability and gait difficulty; UPDRS III, Unified Parkinson’s Disease Rating Scale III; FS-14, 14-item Chalder Fatigue Scale; TNF-α, tumor necrosis factor-α*.

### Comparisons of Neurotransmitters in CSF Between PD-D and PD-ND Groups

The levels of DA, 5-HT, and NE in CSF from PD-D and PD-ND groups were then compared (Table 5). It was observed that DA level in PD-D group was significantly reduced compared with that in PD-ND group. However, the levels of 5-HT and NE were not significantly different between the two groups.

**Table 5:** Levels of neurotransmitters in CSF from PD-D and PD-ND groups; *P < 0.05.

	PD-ND group (28 cases)	PD-D group (58 cases)	*P*
DA (fg/ml, $\bar{\text{X}}$ ± s)	7.6111 ± 2.510	6.232 ± 2.151	0.011*
5-HT (fg/ml, $\bar{\text{X}}$ ± s)	18.459 ± 7.373	14.892 ± 8.367	0.061
NE (fg/ml, $\bar{\text{X}}$ ± s)	502.111 ± 124.634	497.076 ± 125.207	0.865

*DA, dopamine; 5-HT, 5-hydroxytryptamine; NE, norepinephrine*.

### The Relationship Between Neuroinflammatory Factors and Neurotransmitters in CSF From PD-D Group

The correlational analysis between DA and TNF-α level in CSF from PD-D group was further conducted. The results showed that DA level in CSF had a significant and negative correlation with TNF-α level in CSF from PD-D group (*r* = −0.320, *P* = 0.003).

## Discussion

In this study, 63.15% of all patients was diagnosed with PD-D. Compared with PD-ND group, PD-D group had significantly severer motor symptoms, more non-motor symptoms, and poor ADL (Table 1). This is the same with our previous research, which indicated that motor symptoms, PIGD type, anxiety, and fatigue were the significant influencing factors of PD-D (Lian et al., 2018).

Increasing evidences indicated that neuroinflammation featured by the microglial overactivation plays a vital role on the pathogenesis and progression of neurodegenerative disorders, like PD (Streit et al., 1999; Olson and Miller, 2004; Li and Zhang, 2016). Autopsy results from PD brains showed the overactivated microglia in the substantia nigra (McGeer et al., 1988). Microglia, accounting for about 10% of glial cells, are the immune cells in the brain and protect human body against damage through eliminating harmful irritants, pathogens, and dead cells (Cláudio et al., 2013) under normal conditions. However, when brain homeostasis is disturbed by various stimuli, microglia are overactivated and initiate neuroinflammatory process, robustly producing series of neuroinflammatory factors, including TNF-α, IL-1β, IL-6, etc. (Hanisch, 2002; Wilms et al., 2007), as well as a variety of harmful free radicals, such as H_2_O_2_ and NO (Block et al., 2007; Gao et al., 2012). In the PD animal model, it was found that microglial activation resulted in pathogenic changes of α-synuclein, which consequently led to Lewy body formation (Theodore et al., 2008). Neuroinflammation causes DA neuronal death, and the dead neurons release the content, for example, α-synuclein, etc., into extracellular spaces, further evoking microglial activation and progressive DA neurodegeneration. Therefore, neuroinflammation is an important mechanism underlying the development and deterioration of PD (Alcalay, 2016).

Depression was considered to be a microglial disease (Yirmiya et al., 2015). In a mouse model of depression induced by a strong pathogen lipopolysaccharide (LPS), the expression of Toll-like receptor 2 in microglia was evidently increased, while RNA levels of neuroinflammatory factors, such as IL-1β and IL-6, in the cortex and hippocampus were significantly enhanced. Minocycline, a tetracycline antibiotic, significantly reduced the levels of the above neuroinflammatory factors and thus improved depressive symptoms, indicating that depression was closely related to microglial activation. Thus, anti-neuroinflammatory drugs improved depressive symptoms by reducing microglial activation (Henry et al., 2008).

In this investigation, an array of neuroinflammatory factors, including H_2_O_2_, NO, TNF-α, IL-1β, IL-6, and PGE_2_ in CSF from PD patients, were measured and compared. The results demonstrated that TNF-α level in CSF from PD-D group was prominently elevated compared with that from PD-ND group (Table 2). Further analysis indicated that the severer the depressive symptoms, the higher the TNF-α level in CSF (Table 3), and this close correlation was still strong after adjusting the risk factors of PD-D (Table 4). It was confirmed that in PD patients, a variety of pathological processes led to microglial activation, which might contribute to DA neuronal death by releasing cytotoxic inflammatory factors, such as TNF-α, IL-1β, IL-6, and so on. Among all these factors, TNF-α was a strong neuroinflammatory factor involved. It might have a direct damaging effect on DA neurons by activating an intracellular death pathway coupled with its receptor expressed on the cell surface. The neuroinflammatory factor might also stimulate the expression of iNOS within microglial (and possibly astroglial) cells and lead to the production of toxic amounts of NO, H_2_O_2_, etc. In turn, these free radicals could potentiate the expression and release of TNF-α by adjacent microglial cells, thereby amplifying further the inflammatory reaction (Chertoff et al., 2011). Therefore, TNF-α plays an important role in the loss of DA neurons.

TNF-α could be detrimental or protective under different context. TNF-α mediated biological reactions through TNF receptor I and II (TNF-RI and TNF-RII; Montgomery and Bowers, 2012), both of which were homologous single-channel transmembrane glycoproteins with different structures. TNF-α was believed to elicit cytotoxicity and apoptosis when it was combined with TNF-RI, and promote cell survival, proliferation, and protective cellular responses when it was combined with TNF-RII (Hanisch, 2002). These differential effects of TNF-RI and TNF-RII are dependent on cell type, environment, age, and the activation status of those cells (Montgomery and Bowers, 2012). It was also found that there were specific regional differences and dual effects of TNF-α signaling in the brain; for example, TNF-α was a promoter of neurodegeneration in the striatum and a protector of neurodegeneration in the hippocampus (Sriram et al., 2006). Moreover, the effect of TNF-α on brain may be determined by the level and time of expression. Chronic expression of low TNF-α level reduced the nigrostriatal neurodegeneration, while high TNF-α level induced progressive neuronal loss (Chertoff et al., 2011). In PD patients, neuroinflammation plays key roles in the dysfunction and death of DA neurons. It was reported that the levels of both TNF-α and TNF-RI in the striatum and substantia nigra were significantly increased (Mogi et al., 2000; McCoy et al., 2006). TNF-α immunoreactive glial cells were found in the substantia nigra of PD patients but not in those of healthy controls (Boka et al., 1994). Dopaminergic neurons might be sensitive to TNF-α, and the elevated TNF-α may lead to DA neuronal death in PD. In this study, it was found that the higher the level of TNF-α in CSF, the severer the symptoms of depression in PD patients. Hence, we speculated that the elevated TNF-α might be predominantly combined with TNF-RI and produce oxidation–reduction reaction, accordingly playing a significantly negative role on PD-D.

The pathophysiology of PD-D might be related to the changes in neurotransmitter systems (Remy et al., 2005). However, there are very limited studies on the levels of neurotransmitters in CSF from PD-D group. In this investigation, a line of neurotransmitters, including DA, 5-HT, and NE in CSF from PD-D and PD-ND groups, were measured and compared. The results indicated that, compared with 5-HT and NE, DA level in PD-D group was significantly reduced compared with that in PD-ND group, indicating a more important role of DA on PD-D than 5-HT and NE (Lian et al., 2018).

Since neuroinflammatory factor TNF-α and neurotransmitter DA were both highly associated with PD-D, we then asked the question of whether there was a relationship between TNF-α and DA in CSF from the PD-D group. Thus, we finally performed correlational analysis between them, and the results revealed that the higher the TNF-α level, the lower the DA level in CSF from PD-D group, indicating that DA neurons in PD-D brains might be highly susceptible to the damage induced by neuroinflammatory factor TNF-α (McGeer and McGeer, 2008). In the LPS-induced PD animal model, neuroinflammation indicated by microglial overactivation specifically caused permanent damage to DA neurons in the substantia nigra but not to γ-aminobutyric acid neurons in the cortex (Herrera et al., 2000). Here, we inferred that neuroinflammation indicated by the significantly enhanced TNF-α in CSF might cause serious damage to DA neurons and consequent dramatic depletion of DA, which was correlated to the occurrence and progression of PD-D.

This study has the following limitations. It was difficult to obtain CSF from all PD patients due to old age, hyperostosis, etc. Currently, we are collecting CSF from PD patients with the aim of confirming the results from the current study. Because an observational study is limited for drawing final conclusions, a longitudinal study is being performed to figure out the correlations among depression, the levels of neuroinflammatory factors, and neurotransmitters in CSF from PD patients for a prolonged period.

## Data Availability Statement

The raw data supporting the conclusions of this article will be made available by the authors, without undue reservation.

## Ethics Statement

The studies involving human participants were reviewed and approved by Beijing Tiantan Hospital ethics committee. The patients/participants provided their written informed consent to participate in this study.

## Author Contributions

T-hL: drafted the manuscript, study design, analysis of data, accepts responsibility for conduct of research, gave final approval, acquisition of data, and statistical analysis. PG: analysis of data, acquisition of data, accepts responsibility for the conduct of research, and gave final approval. Y-nZ, W-jZ, H-yG, and X-mW: accepts responsibility for the conduct of research, and gave final approval. J-hL: assisted in drafting the manuscript, accepts responsibility for the conduct of research, and gave final approval. L-xL, D-yD, and D-nL: acquisition of data, accepts responsibility for the conduct of research, and gave final approval. W-jZ: study design, analysis of data, accepts responsibility for the conduct of research, gave final approval, acquisition of data, statistical analysis, and study supervision.

## Conflict of Interest

The authors declare that the research was conducted in the absence of any commercial or financial relationships that could be construed as a potential conflict of interest.
